# Low bone mineral density is a prognostic factor for elderly patients with HCC undergoing TACE: results from a multicenter study

**DOI:** 10.1007/s00330-022-09069-8

**Published:** 2022-08-20

**Authors:** Lukas Müller, Aline Mähringer-Kunz, Timo Alexander Auer, Uli Fehrenbach, Bernhard Gebauer, Johannes Haubold, Jens M. Theysohn, Moon-Sung Kim, Jens Kleesiek, Thierno D. Diallo, Michel Eisenblätter, Dominik Bettinger, Verena Steinle, Philipp Mayer, David Zopfs, Daniel Pinto dos Santos, Roman Kloeckner

**Affiliations:** 1 Department of Diagnostic and Interventional Radiology, University Medical Center of the Johannes Gutenberg University Mainz, Langenbeckst. 1, 55131 Mainz, Germany; 2 Department of Radiology, Charité – University Medicine Berlin, Berlin, Germany; 3 Department of Diagnostic and Interventional Radiology and Neuroradiology, University Hospital Essen, Essen, Germany; 4 Institute for AI in Medicine (IKIM), University Hospital Essen, Essen, Germany; 5 Department of Diagnostic and Interventional Radiology, Freiburg University Hospital, Freiburg, Germany; 6 Department of Medicine II, Medical Center University of Freiburg, Faculty of Medicine, University of Freiburg, Freiburg, Germany; 7 Department of Diagnostic and Interventional Radiology, University Medical Center Heidelberg, Heidelberg, Germany; 8 Department of Radiology, University Hospital Cologne, Cologne, Germany; 9 Department for Interventional Radiology, University Hospital of Lübeck, Ratzeburger Allee 160, Lübeck, Germany

**Keywords:** Hepatocellular carcinoma, Transarterial chemoembolization, Bone mineral density, Prognosis

## Abstract

**Objectives:**

Low bone mineral density (BMD) was recently identified as a novel risk factor for patients with hepatocellular carcinoma (HCC). In this multicenter study, we aimed to validate the role of BMD as a prognostic factor for patients with HCC undergoing transarterial chemoembolization (TACE).

**Methods:**

This retrospective multicenter trial included 908 treatment-naïve patients with HCC who were undergoing TACE as a first-line treatment, at six tertiary care centers, between 2010 and 2020. BMD was assessed by measuring the mean Hounsfield units (HUs) in the midvertebral core of the 11^th^ thoracic vertebra, on contrast-enhanced computer tomography performed before treatment. We assessed the influence of BMD on median overall survival (OS) and performed multivariate analysis including established estimates for survival.

**Results:**

The median BMD was 145 HU (IQR, 115–175 HU). Patients with a high BMD (≥ 114 HU) had a median OS of 22.2 months, while patients with a low BMD (< 114 HU) had a lower median OS of only 16.2 months (*p* < .001). Besides albumin, bilirubin, tumor number, and tumor diameter, BMD remained an independent prognostic factor in multivariate analysis.

**Conclusions:**

BMD is an independent predictive factor for survival in elderly patients with HCC undergoing TACE. The integration of BMD into novel scoring systems could potentially improve survival prediction and clinical decision-making.

**Key Points:**

*• Bone mineral density can be easily assessed in routinely acquired pre-interventional computed tomography scans.*

*• Bone mineral density is an independent predictive factor for survival in elderly patients with HCC undergoing TACE.*

*• Thus, bone mineral density is a novel imaging biomarker for prognosis prediction in elderly patients with HCC undergoing TACE.*

**Supplementary Information:**

The online version contains supplementary material available at 10.1007/s00330-022-09069-8.

## Introduction

Hepatocellular carcinoma (HCC) is the most common primary liver cancer and is a leading cause of cancer-related deaths worldwide [1]. Guidelines from both the European Association for the Study of the Liver (EASL) and the American Association for the Study of Liver Diseases (AASLD) recommend the use of the Barcelona Clinic Liver Cancer (BCLC) classification system as a framework for patient stratification, treatment allocation, and prognosis prediction in patients with HCC [2, 3]. According to the BCLC classification, transarterial chemoembolization (TACE) is the treatment of choice for patients with intermediate-stage HCC [4]. However, in clinical settings, intermediate-stage disease includes a heterogeneous group of patients with broad variations in tumor load and remaining liver function [5]. Furthermore, TACE is administered to patients within other BCLC stages, either following the concept of stage migration or based on individual treatment decisions [6]. Thus, it is exceptionally difficult to perform risk scoring and prognosis prediction among patients treated with TACE [7].

One problem with conventional scoring systems is their focus on factors mainly based on tumor burden and remaining liver function. To address this issue, novel approaches have been designed to construct a more holistic view of patients by accounting for additional factors, like immunonutrition [8]. Additionally, recent technical developments in artificial intelligence-based automized risk calculation constitute a basis for the broad integration of additional risk factors in daily treatment decision-making [9].

For patients with HCC, the quantification of bone mineral density (BMD) as a surrogate for osteopenia may be a novel and promising prognostic factor. BMD is conventionally assessed using dual-energy x-ray absorptiometry (DXA) or quantitative computed tomography (QCT); however, these methods entail additional costs and radiation exposure [10]. Image data routinely acquired during diagnostic work-up can be used for a simple and ubiquitously available method of BMD assessment. Osteopenia, assessed using this simple method of BMD assessment, has already been identified as a prognostic factor for patients undergoing tumor resection or liver transplantation [11–13]. However, pretreatment measurement of BMD has not been investigated as a potential risk factor in patients undergoing TACE.

In the present multicenter study, we aimed to evaluate the role of osteopenia in the outcome of patients with HCC undergoing TACE.

## Material and methods

This study protocol was approved by the Ethics Committee of the Medical Association of Rhineland-Palatinate, Germany (permit number 15913). The requirement for informed consent was waived due to the retrospective nature of the study. All other locally responsible ethics committees followed this decision. Patient records and information were anonymized at the local centers prior to data transfer. This report follows the guidelines for transparent reporting of a multivariable prediction model for individual prognosis or diagnosis (TRIPOD) [14].

### Patients

A total of six German tertiary care centers participated. The included patients met the following criteria: (1) first TACE between January 2010 and December 2020 to allow a minimum follow-up of 6 months; (2) age above 18 years; (3) histologically or image-derived HCC diagnosis based on the EASL criteria; (4) no treatment performed prior to TACE; (5) no liver transplantation or tumor resection during the follow-up period after TACE (such that all included patients underwent TACE performed with palliative intent); (6) within BCLC stages 0, A, B, or C; (7) CT scan including the 11th vertebra for BMD calculation; and (8) full availability of clinical, laboratory, and imaging data (Supplementary Fig. S1).

### Diagnosis, treatment, and data acquisition

HCC was diagnosed based on histological or image-derived EASL criteria, as previously reported [2, 15]. Prior to their first TACE treatment, all patients underwent contrast-enhanced CT for diagnosis, staging, and treatment planning. TACE was performed in a standardized manner, as previously described in detail [16–18]. Follow-up comprised clinical examination, blood sampling, and cross-sectional imaging. The primary end-point was median overall survival (OS), defined as the time interval between the initial TACE session and death or last follow-up. All baseline characteristics, including demographic data, liver disease status, and etiology, as well as TACE-related parameters and laboratory parameters, were obtained from each hospital information system and from the laboratory database. Information regarding the tumor burden—including tumor growth pattern, number of lesions, and diameter of the largest target lesion—were determined based on the radiological report and the cross-sectional images.

### BMD assessment

**Fig. 1:**
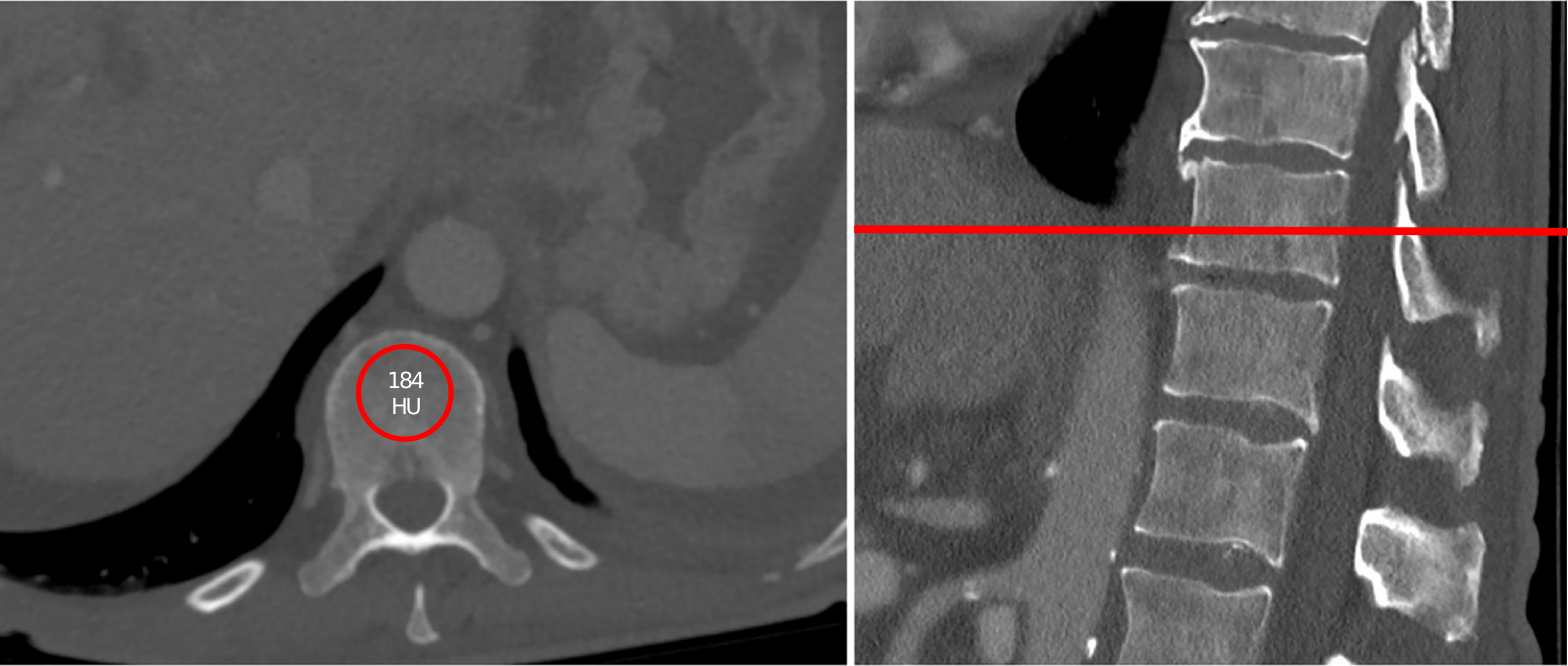
Example for the measurement of the bone mineral density (BMD)

BMD was assessed by measuring the Hounsfield units (HUs) of the 11^th^ thoracic vertebra during the venous phase on contrast-enhanced CT prior to treatment, as previously reported [12, 13]. For this measurement, a region-of-interest (ROI) with a diameter of 10–15 mm was placed in the trabecular midvertebral core, cranial to the base plate of the vertebral body, in a standardized manner (Fig. 1).

For quality control, each center received precise written instructions prior to the start of measurements. To further minimize bias, a training session was conducted with all participants. Throughout the entire study, upcoming questions were discussed in a weekly progress meeting. The optimal cut-off for stratifying patients into low and high BMD groups was 114 HU. In cases with bone lesions, compression fractures, or image artifacts, BMD was assessed in the vertebra above or below the 11^th^ thoracic vertebra (*n* = 3, 0.3%).

### Statistical analysis

Statistical analyses and graphic design were performed in R 4.0.3 (A Language and Environment for Statistical Computing, R Foundation for Statistical Computing, http://www.R-project.org; last accessed May 15, 2022). Continuous data were reported as median and interquartile range. Categorical and binary baseline parameters were reported as absolute numbers and percentages. Categorical parameters were compared using Fisher’s exact test, and continuous parameters using the Mann-Whitney test. Correlation coefficients were calculated according to Pearson. The optimal BMD cut-off was determined using optimal stratification methodology, with the packages “survminer” and “survival” (https://cran.r-project.org/package=survminer, https://CRAN.R-project.org/package=survival, accessed May 15, 2022). These packages were also used for survival analyses. Univariate and multivariate Cox proportional hazards regression models were used to determine the effect of the risk stratification, and to evaluate the roles of included factors, with the findings reported as hazard ratios (HRs) and corresponding 95% confidence intervals (CIs). A *p* value of < 0.05 was considered significant in all tests.

## Results

### Baseline characteristics

Table 1 presents all baseline characteristics at the initial TACE treatment.

**Table 1:** Baseline characteristics of the included patients.

Variable	All patients (*n* = 908)
Age in years, median (IQR)	67 (60–75)
Sex, *n* (%)	
Female	176 (19.4)
Male	732 (80.6)
Etiology, *n* (%)	
No cirrhosis	103 (11.3)
Alcohol	360 (39.7)
Viral	270 (29.7)
Other	175 (19.3)
Child-Pugh stage, *n* (%)	
No cirrhosis	103 (11.3)
A	499 (55.0)
B	306 (33.7)
BCLC stage, *n* (%)	
0	14 (1.5)
A	272 (30.0)
B	490 (54.0)
C	132 (14.5)
Size of the largest lesion in mm, max (IQR)	40 (27–64)
Number of lesions, median (IQR)	2 (1–4)
Albumin level, median (IQR)	36 (31–40)
Bilirubin level, median (IQR)	1.1 (0.7–1.8)
Platelet count, median (IQR)	127 (85–192)
AST level, median (IQR)	60 (41–91)
ALT level, median (IQR)	40 (27–62)
INR, median (IQR)	1.1 (1.1–1.2)
AFP level, median (IQR)	20.9 (5.7–348.5)

*BCLC*, Barcelona Clinic Liver Cancer; *AST*, aspartate aminotransferase; *ALT*, alanine aminotransferase; *AFP*, alpha fetoprotein

**Fig. 2:**
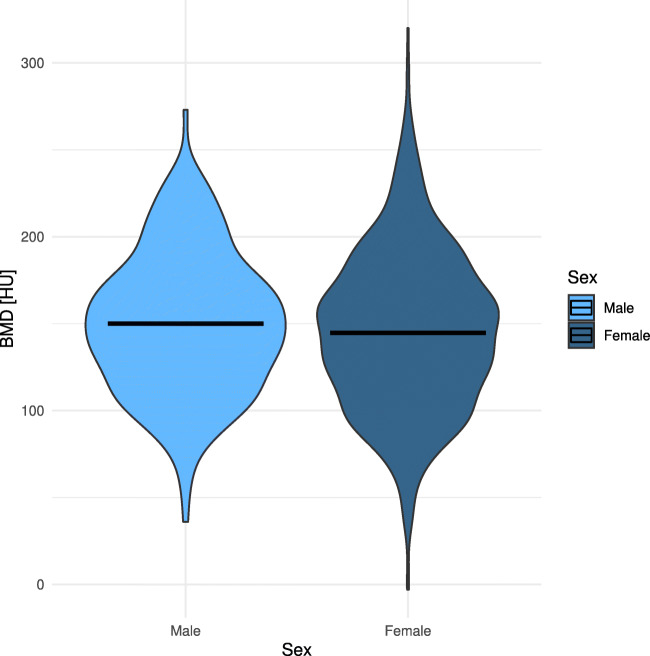
Distribution of bone mineral density (BMD) among males and females

The median BMD of the entire patient cohort was 145 HU. The median BMD did not significantly differ between male and female patients (145.0 and 150.0, *p* = .224) (Fig. 2).

### Influence of BMD on survival after TACE

**Fig. 3:**
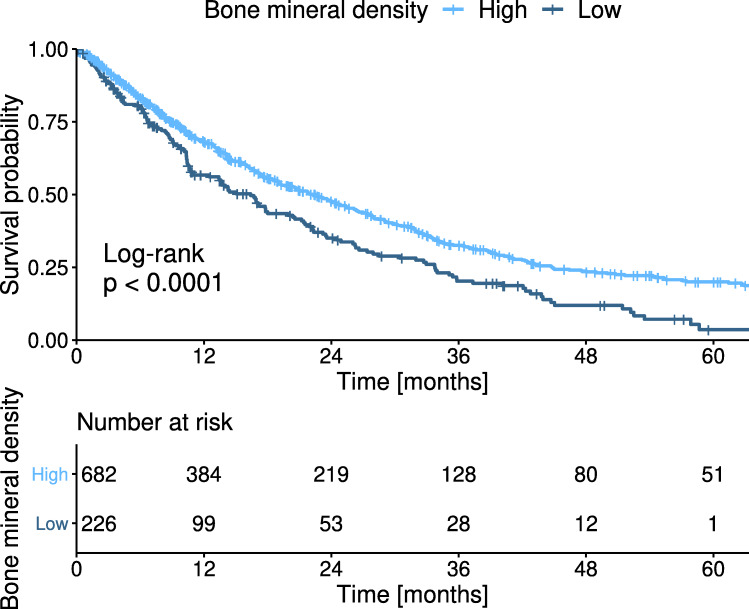
Kaplan-Meier curves for the overall survival of patients stratified according to their bone mineral density (BMD)

Patients with a high BMD (≥ 114 HU) had a median OS of 22.2 months. Patients with a low BMD (< 114 HU) had a significantly lower median OS of 16.2 months (*p* < .001) (Fig. 3).

Subgroup analysis was performed among 490 (50.8%) patients with BCLC stage B (i.e., the recommended TACE subgroup) [2, 3]. Using the same cut-off, the patients with a high BMD (≥ 114 HU) had a median OS of 18.9 months, while patients with a low BMD (< 114 HU) had a significantly lower median OS of 16.5 months (*p* = .029) (Supplementary Fig. S2).

Because of the relatively high median age in the overall cohort, we performed a subgroup analysis including patients in the first age quartile (≤ 60 years). Using the same cut-off of 114 HU in this subgroup, patients with a low BMD had a median OS of 16.2 months, while patients with a high BMD had a median OS of 19.0 months (*p* = .036) (Supplementary Fig. S3).

Apart from stratification using the optimal cut-off value, we also assessed survival with stratification using cut-off values of 160 HU and 110 HU, which have yielded the highest values of sensitivity and specificity, respectively, for osteoporosis detection. Both cut-off values yielded a significant stratification (Supplementary Fig. S4).

### The role of BMD as an independent predictor of survival

In a second step, we performed regression analysis to assess whether BMD is an independent prognostic factor for OS after TACE. In univariate regression analysis, the covariates albumin, bilirubin, AST, INR, focality, and tumor diameter were identified as prognostic factors for median OS, in addition to BMD (Table 2). In the multivariate regression analysis, only albumin, bilirubin, tumor diameter, tumor number, and BMD remained independent prognostic factors.

**Table 2:** Univariate and multivariate cox regression analysis

Analysis		Univariate			Multivariate		
Covariate		HR	95% CI	*p* value	HR	95% CI	*p* value
Age	≥ 70 years	0.8	0.7–1.0	0.060			
AFP	> 400 ng/mL	1.0	0.8–1.2	0.940			
Albumin	< 35 g/L	2.0	1.7–2.4	< 0.001	1.9	1.5–2.3	< 0.001
Bilirubin	≥ 1.2 mg/dL	1.8	1.5–2.1	< 0.001	1.6	1.3–2.0	< 0.001
AST level	> 31 U/L	1.5	1.1–2.0	0.011	1.3	0.9–1.9	0.148
ALT level	≥ 35 U/L	1.1	0.9–1.3	0.570			
INR level	> 1.2	1.3	1.1–1.6	0.003	1.0	0.8–1.3	0.997
Platelet count	< 150/nL	1.1	0.9–1.3	0.470			
Tumor number	≥ 2	1.4	1.2–1.7	< 0.001	1.3	1.0–1.6	0.027
Max. lesion size	> 5.0 cm	1.4	1.2–1.7	< 0.001	1.7	1.4–2.0	< 0.001
BMD	< 114 HU	1.4	1.2–1.7	< 0.001	1.7	1.4–2.1	< 0.001

HR, hazard ratio; *CI*, confidence interval; *AFP*, alpha fetoprotein; *AST*, aspartate aminotransferase; *ALT*, alanine aminotransferase; *BMD*, bone mineral density

To further evaluate the role of BMD as an independent predictor, we next assessed whether BMD was correlated with factors related to liver function and tumor burden. The determined correlation coefficients ranged between −0.140 and 0.170 (Table 3, Supplementary Fig. S5). Values of < 0.3 indicate a small correlation [19]. The correlation coefficient between age and BMD was −0.370 (*p* < .001), indicating a weak-to-moderate correlation [19].

**Table 3:** Correlation between BMD and surrogates of liver function and tumor burden

Parameter	Correlation coefficient	*p* value
Liver function		
*Albumin*	−0.038	0.280
*Bilirubin*	0.130	< 0.001
*Thrombocytes*	−0.150	< 0.001
*INR*	0.100	0.002
Tumor burden		
*Largest tumor diameter*	−0.066	0.057
*Number of tumors*	−0.038	0.280

**Fig. 4:**
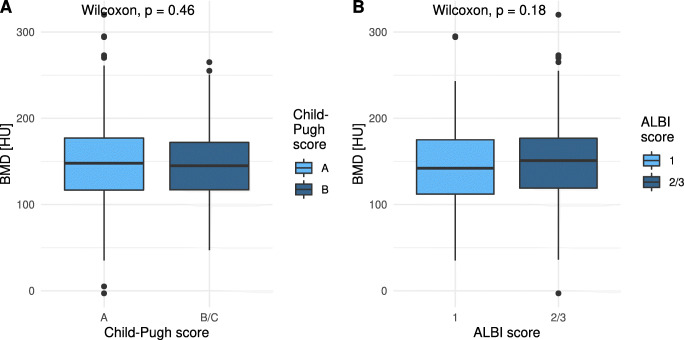
Bone mineral density (BMD) according to Child-Pugh and ALBI score stage. Distribution of the BMD among various (**A**) Child-Pugh stages and (**B**) ALBI score stages

Additionally, BMD did not significantly differ among patients with different Child-Pugh and ALBI score stages (Fig. 4). Furthermore, BMD did not significantly differ among the various BCLC stages (Supplementary Fig. S6 and Supplementary Table S1).

## Discussion

In this study, we investigated the role of BMD as a potential novel imaging biomarker for patients with HCC who are undergoing TACE. In summary, we demonstrated that BMD was a highly predictive and independent prognostic factor for survival.

To date, few studies have investigated the role of BMD in patients with HCC who are undergoing curative treatment [11, 12, 20]. Low BMD before liver transplantation has been identified as an independent prognostic factor for post-transplant mortality [11, 13]. Moreover, BMD is a highly predictive factor for patients with HCC who are undergoing resection [12]. However, BMD has not previously been evaluated in patients with HCC who are undergoing TACE. In a pilot study, Zheng et al investigated the role of the change in BMD during treatment as a potential risk factor. Among 75 Asian patients undergoing TACE, they observed a median BMD change of −8.7% per 120 days [21]. However, 33 (44%) of these patients exhibited an increased BMD after their first TACE. Interestingly, the patients who showed BMD loss after TACE had a lower initial BMD compared to patients who exhibited a BMD increase after TACE. Median OS did not differ between patients who showed a BMD gain versus loss after their first TACE. However, that trial did not investigate the prognostic influence of the absolute BMD prior to treatment.

Another previous report demonstrates that TACE is associated with a higher rate of osteoporosis [22]. The authors concluded that the cumulative radiation dose might be a factor contributing to the higher prevalence of osteoporosis in those patients. However, they did not validate their results in a group of patients with HCC who did not undergo TACE. Thus, it remains unclear whether the presence of HCC itself, as a highly wasting tumor disease, contributed to the higher prevalence of osteopenia in these patients. Future studies on this subject should further explore the role of treatment-related BMD changes. It would also be useful to identify factors associated with a low BMD prior to treatment, and with a BMD decrease during treatment, since those factors might be targets for specific pre-treatment interventions.

In a multivariate Cox regression analysis, apart from BMD, liver function and tumor size were identified as independent prognostic factors of OS. Remaining liver function and tumor burden are well-known risk factors and play the most important role in conventional scoring systems for predicting the prognosis of patients with HCC undergoing TACE [7]. Interestingly, BMD showed only weak correlations with liver function-related laboratory parameters and with tumor size, as reflected by correlation coefficients ranging from −0.130 to 0.140. These results are in line with previous reports in patients with HCC, in which BMD did not correlate with the remaining liver function as well [12, 13]. Thus, BMD can be considered an independent prognostic factor. This suggests that BMD could function as an additional parameter in interdisciplinary decision-making, along with the conventional risk factors of liver function and tumor burden. BMD as well as other risk factors related to the patient’s constitution could help to build a more holistic picture of the patients, subsequently leading to improved individual patient selection. Recent approaches show that the inclusion of multiple factors could become more easily in daily routine using novel methods including deep learning and that such approaches might outperform conventional scoring systems [9]. Thus, we believe that the identification of novel risk factors has a high potential to further improve patient selection and therefore lead to significant proceedings in the field. Notably, given the heterogeneity of patients with HCC, BMD can only be one of several components in decision-making, and a thorough interdisciplinary discussion is mandatory. Previous studies in healthy patients and in other tumor entities have identified a correlation of BMD with age. In our study, BMD and age showed only a weak-to-moderate correlation, similar to previous reports on patients with HCC undergoing curative treatment [12, 13]. These different observations might be explained by the co-existence of two chronic diseases, namely HCC and liver cirrhosis, in our patient cohort. Both diseases lead to substantial changes in the patient’s constitution and have a dominant impact on bone metabolism. Therefore, in this population, BMD might be more strongly related to the duration and severity of both diseases than to the patient’s age.

Regarding the technique of BMD assessment, previous studies have clearly shown that routine CT scans allow for reliable opportunistic BMD screening. Pickhardt et al compared BMD values obtained from DXA and CT in patients who underwent a CT colonography within a two-month period around the time of DXA [23]. Overall, BMD determined from non-enhanced abdominal CT scans was highly correlated with the values obtained from DXA and, therefore, reached high predictive values for osteoporosis detection. However, in clinical reality, many CT scans are performed without a pre-contrast native phase. In these cases, only contrast-enhanced CT scans are available for BMD assessment. Although intravenous contrast media can reach the trabecular region of the vertebra, the observed effect on enhancement of this region is minimal and does not impact the ability to assess BMD based on thoracolumbar vertebras [10, 24]. In the general population, a cut-off of < 100 HU has been reported as highly specific for osteoporosis diagnosis, while a cut-off of 150 HU yields the highest sensitivity for osteoporosis detection [25]. In our study, the optimal cut-off for survival stratification was 114 HU, which is between these previously determined values. In the future, it may be possible to fully automate BMD assessment using AI-based methods, and the quantified BMD value could become part of standardized radiologic reporting. Such an approach would facilitate the combination of BMD with other body composition parameters. To foster integration into standard clinical workflows, we must standardize the utilized body composition parameters and BMD, and determine reference values.

In this study, we decided to measure the BMD at the level of the 11^th^ vertebra. This height of the spine is covered in patients receiving abdominal CT. Notably, the 11^th^ vertebra is also covered in thoracic CT, which is part of the standard work-up for staging if patients undergo a liver MRI as the initial imaging modality. Thus, BMD measurement from the 11^th^ vertebra can be performed for every patient with HCC and undergoing TACE.

In this study, we chose overall survival as the primary endpoint. Our patient recruitment ranged from 2010 to 2020. During this period, treatment options after TACE ineligibility were largely limited to the use of the tyrosine-kinase inhibitor sorafenib. However, in clinical reality, few patients received any systemic treatment after TACE failure [26]. With the use of novel immunotherapeutic treatment options after TACE ineligibility, OS evaluation may become a suboptimal endpoint for studies investigating TACE, since more patients could be eligible for an early switch to potent systemic treatment after TACE failure. Such a change of care might substantially influence post-TACE survival in the future. Nevertheless, no robust evidence supports alternative endpoints in trials for liver cancer, and particularly for patients undergoing TACE. PFS and TTP might be too vulnerable to cover the real clinical situation of these patients [27]. Alternative composite endpoints (e.g., “failure of strategy” (NCT04803994)) are currently used in clinical trials in this field but are not reconstructable in a retrospective setting. There remains a need for future studies investigating alternative endpoints for liver trials, and their correlation to survival as the only endpoint with “absolute precision” [27].

Our present study has several limitations. First, it had a retrospective design. Therefore, it would be useful to verify the present results in a prospective setting. Second, our study cohort included patients within BCLC stages 0, A, B, and C. Although TACE is the recommended standard treatment for patients with intermediate-stage disease (BCLC stage B), our cohort reflects the clinical reality in most countries. TACE is commonly applied in more advanced or earlier stages, within the concept of stage migration, which has been endorsed by the EASL [2]. Thus, the heterogeneity of our cohort may hamper the generalizability of our results and should be considered a limitation. Notably, we performed a subgroup analysis among the patients with BCLC stage B, for whom TACE is the recommended standard treatment, and found that BMD remained an independent prognostic factor. Third, in our study, BMD was assessed manually. However, in the future, BMD will be easily assessable through automated measurement using novel artificial intelligence (AI) techniques. Furthermore, BMD could become potentially one surrogate for the overall patient status in more precise and individualized risk classification using the above-mentioned AI methods. Fourth, BMD was measured for only one vertebra. However, this method facilitates a fast and highly reproducible measurement that would be practical for clinical routine. Notably, in the future, manual measurements may be replaced by fully automated BMD assessments from all routinely acquired CT scans. Such measurements could be easily integrated into structured radiologic reports without any additional effort. Fifth, we did not perform any subgroup analysis of patients treated using different TACE techniques. However, multiple comparisons between cTACE and DEB-TACE have not revealed any influence on OS [28–30].

## Conclusion

Low BMD is an independent predictive factor for survival in elderly patients with HCC who are undergoing TACE. The integration of BMD into novel scoring systems could potentially improve survival prediction and clinical decision-making.

## Supplementary information

ESM 1 (PDF 564 kb)

## Abbreviations

ALBI: Albumin-bilirubin
AFP: Alpha fetoprotein
AASLD: American Association for the Study of Liver Diseases
AST: Aspartate aminotransferase
BMD: Bone mineral density
CT: Computed tomography
CIs: Confidence intervals
DXA: Dual-energy x-ray absorptiometry
EASL: European Association for the Study of the Liver
HRs: Hazard ratios
HBV: Hepatitis B virus
HCC: Hepatocellular carcinoma
HUs: Hounsfield units
OS: Overall survival
QCT: Quantitative computed tomography
ROI: Region of interest
TACE: Transarterial chemoembolization

## Declarations

### Guarantor

The scientific guarantor of this publication is Professor Dr. med. Roman Kloeckner.

### Statistics and biometry

One of the authors has significant statistical expertise.

### Methodology

• retrospective

• diagnostic or prognostic study

• multicenter study
