# Supplementary material for: Low bone mineral density is a prognostic factor for elderly patients with HCC undergoing TACE: results from a multicenter study
Source: Eur Radiol. 2022 Aug 20;33(2):1031–9. doi: 10.1007/s00330-022-09069-8 (PMC9889510; doi:10.1007/s00330-022-09069-8)
Supplement: Supplementary file 1 — (PDF 564 kb) [file 330_2022_9069_MOESM1_ESM.pdf]

Fig. S1 Flowchart showing patient inclusion and the final number of patients for whom BMD could be evaluated.

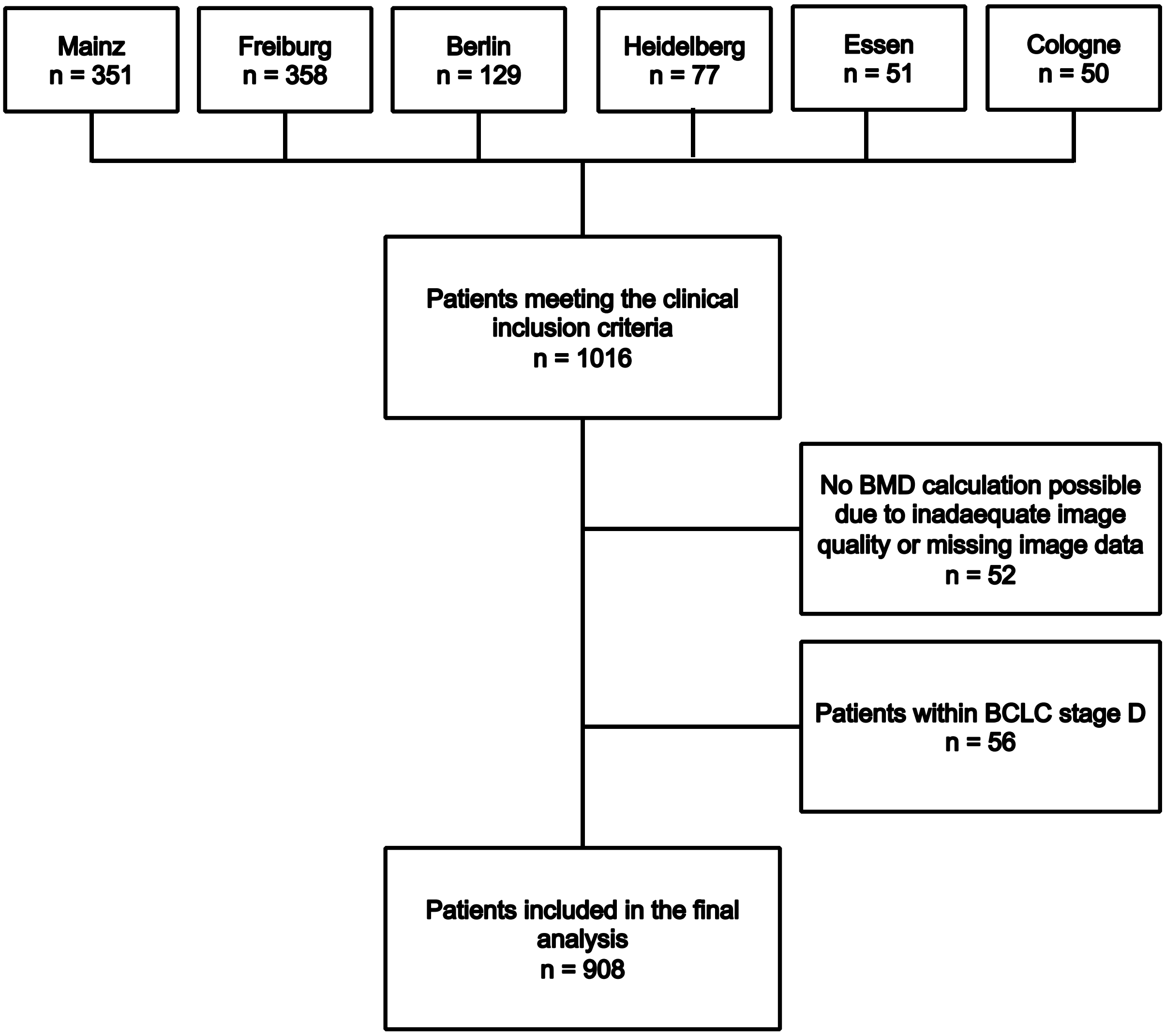

Fig. S2. Kaplan-Meier curves for the overall survival of patients with BCLC stage B (n = 490), stratified according to their bone mineral density (BMD).

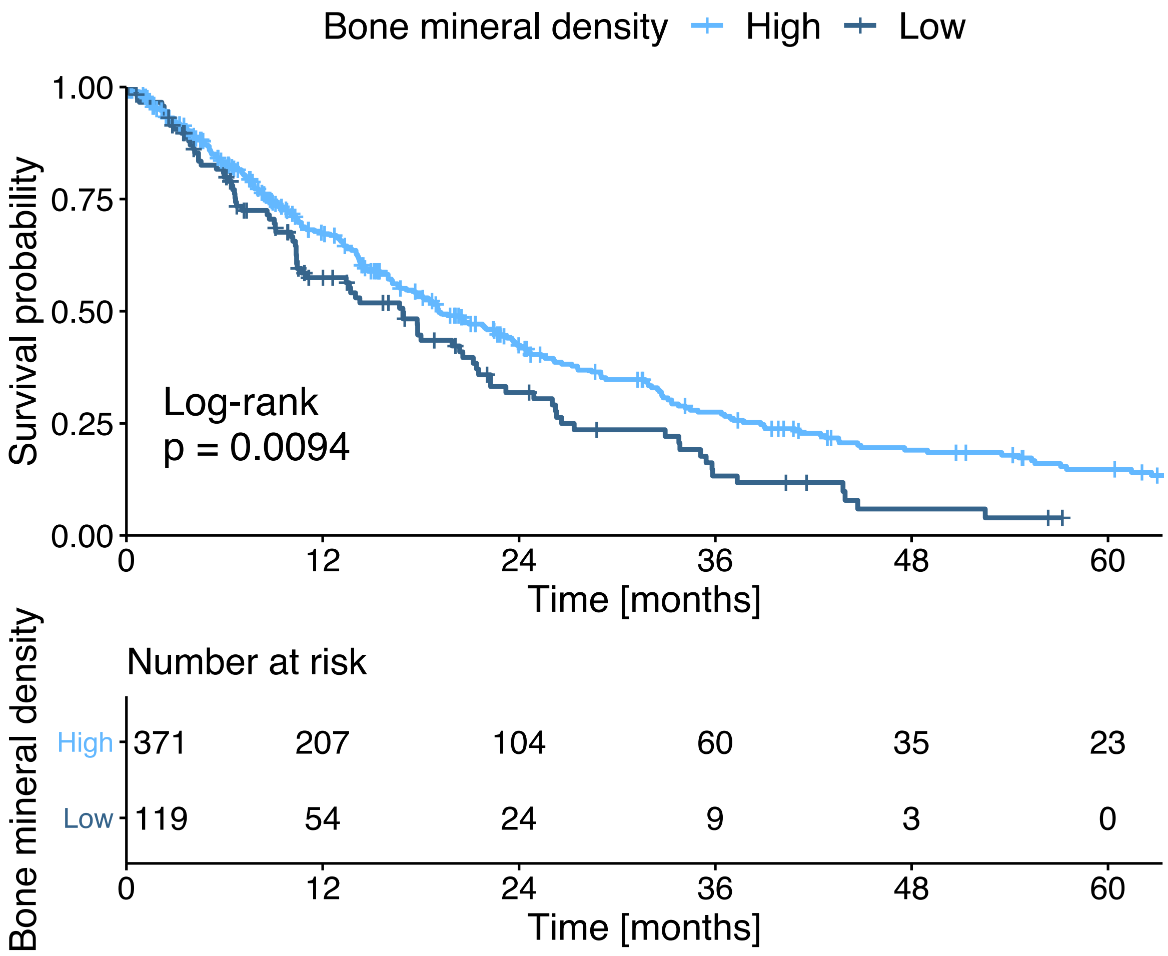

Fig. S3. Kaplan-Meier curves for the overall survival of patients younger than 60 years (n = 245), stratified according to their bone mineral density (BMD).

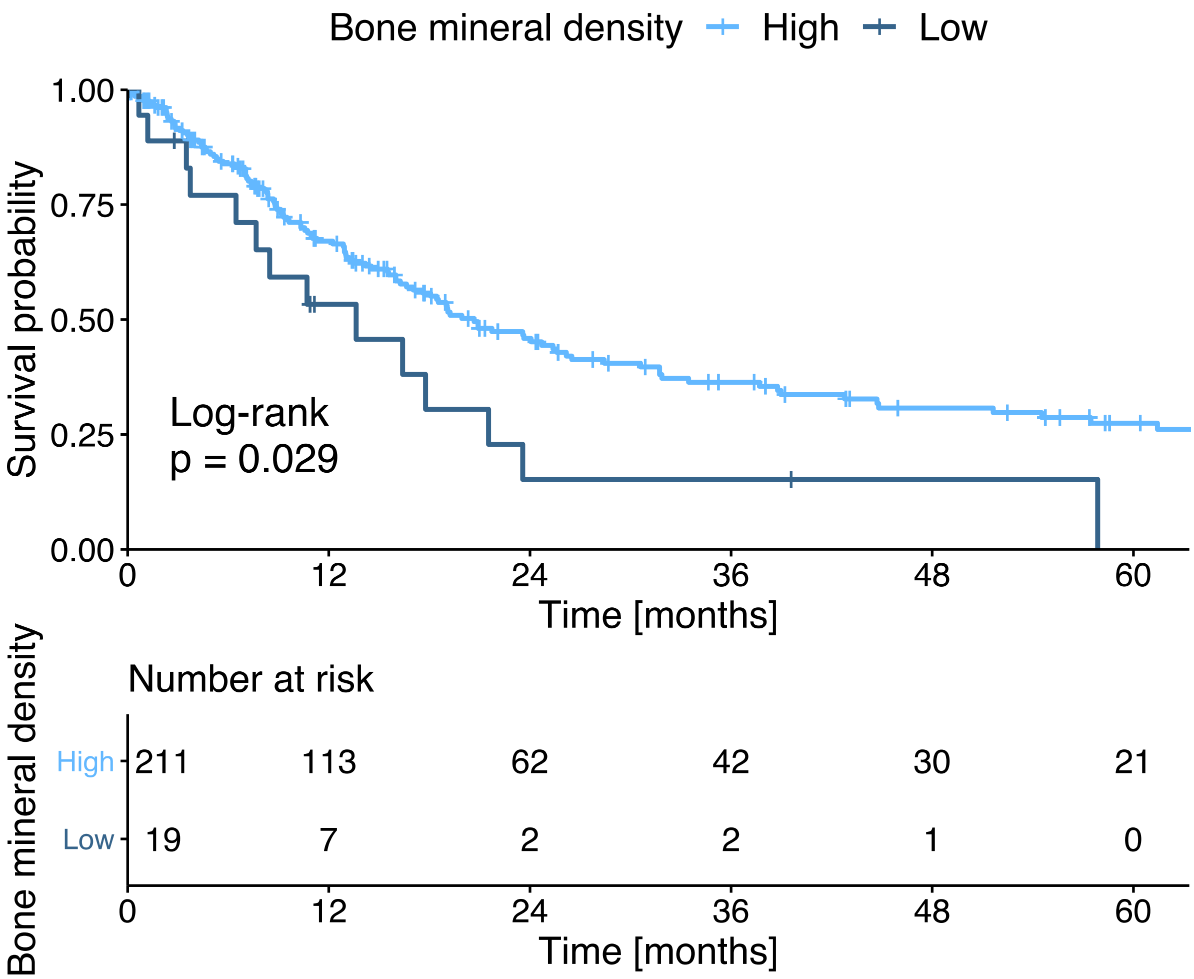

Fig. S4. Kaplan-Meier curves for overall survival. Overall survival of patients stratified according to their bone mineral density (BMD), with cut-off values of (A) 160 HU and (B) 110 HU.

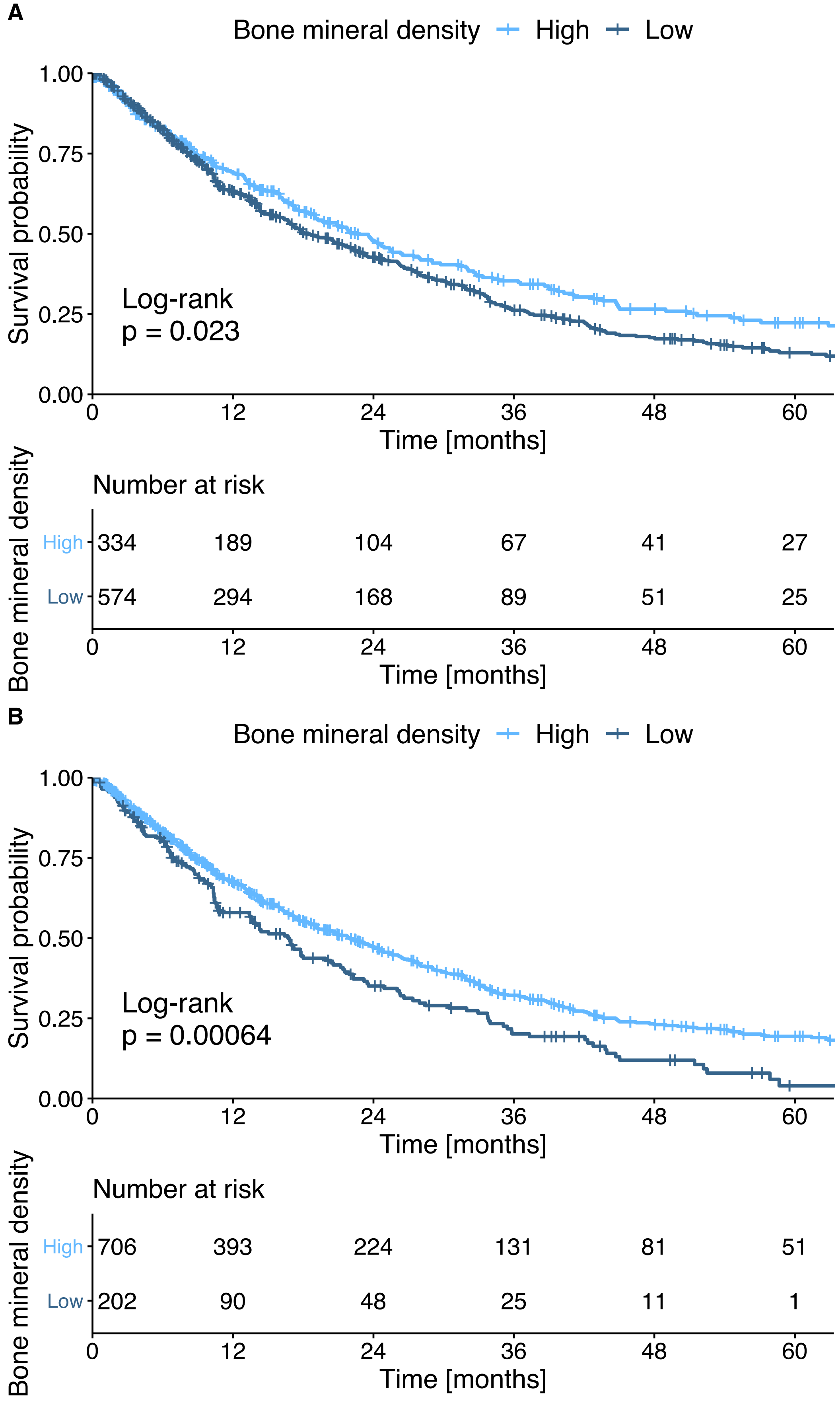

Fig. S5. Correlations of bone mineral density (BMD) with various parameters. Correlation of BMD with albumin (A), bilirubin (B), platelet count (C), INR (D), largest tumor diameter (E), and number of lesions (F).

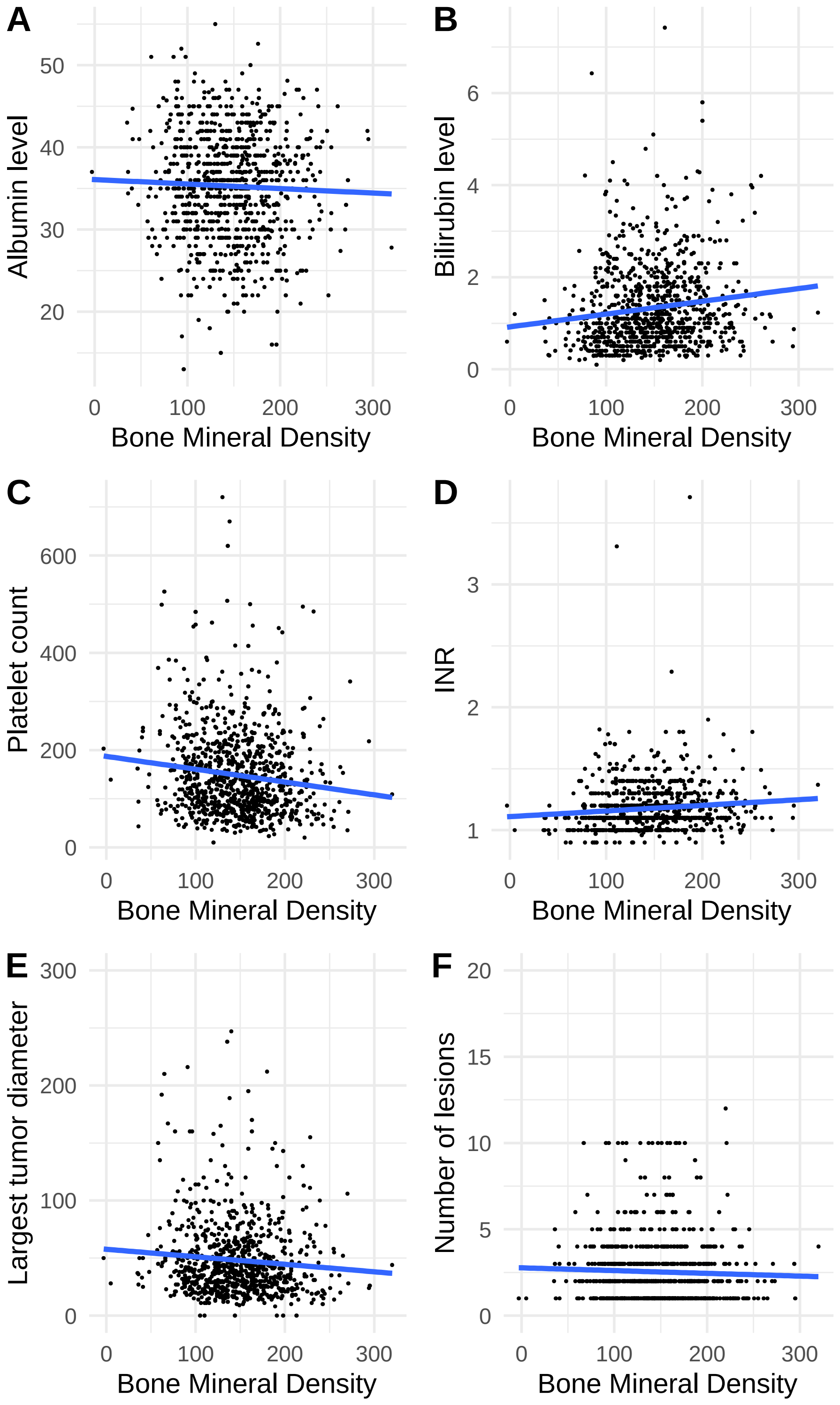

Fig. S6. Bone mineral density (BMD) according to the BCLC stages.

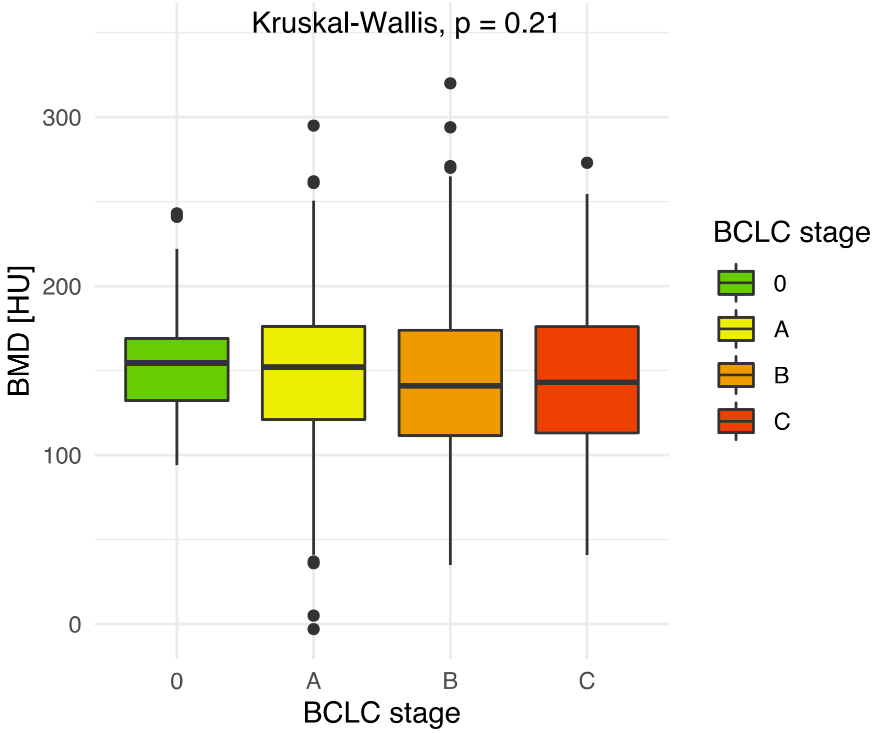

Supplementary Table S1. Pairwise comparisons of the BMD among various BCLC stages (p values).

**BCLC stage**	**0**	**A**	**B**	**C**
**0**	-	0.570	0.260	0.320
**A**	0.570	-	0.061	0.310
**B**	0.260	0.061	-	0.630
**C**	0.320	0.310	0.630	-
